# Hypermutated phenotype in gliosarcoma of the spinal cord

**DOI:** 10.1038/s41698-021-00143-w

**Published:** 2021-02-12

**Authors:** Christopher S. Hong, Gregory A. Kuzmik, Adam J. Kundishora, Aladine A. Elsamadicy, Andrew B. Koo, Declan McGuone, Nicholas A. Blondin, Michael L. DiLuna, E. Zeynep Erson-Omay

**Affiliations:** 1 Department of Neurosurgery, Yale School of Medicine, New Haven, CT 06511 USA; 2 Department of Pathology, Yale School of Medicine, New Haven, CT 06511 USA; 3 Department of Neurology, Yale School of Medicine, New Haven, CT 06511 USA

**Keywords:** Surgical oncology, Cancer genomics

## Abstract

Gliosarcoma is a variant of glioblastoma with equally poor prognosis and characterized by mixed glial and mesenchymal pathology. Metastasis is not uncommon but the involvement of the spinal cord is rare, and comprehensive genetic characterization of spinal gliosarcoma is lacking. We describe a patient initially diagnosed with a low-grade brain glioma via biopsy, followed by adjuvant radiation and temozolomide treatment. Nearly 2 years after diagnosis, she developed neurological deficits from an intradural, extramedullary tumor anterior to the spinal cord at T4, which was resected and diagnosed as gliosarcoma. Whole-exome sequencing (WES) of this tumor revealed a hypermutated phenotype, characterized by somatic mutations in key DNA mismatch repair (MMR) pathway genes, an abundance of C>T transitions within the identified somatic single nucleotide variations, and microsatellite stability, together consistent with temozolomide-mediated hypermutagenesis. This is the first report of a hypermutator phenotype in gliosarcoma, which may represent a novel genomic mechanism of progression from lower grade glioma.

## Introduction

Gliosarcomas represent a variant of glioblastoma, characterized by mixed histopathological features of glial and mesenchymal origins^1^. Similar to glioblastoma, patients diagnosed with gliosarcoma exhibit poor prognosis and overall survival^2^. Metastasis is relatively common within the central nervous system (CNS), as well as systemic visceral sites^1^. Gliosarcomas involving the spinal cord are rare, making up approximately 1% of all malignant gliomas of the spine^3^ and may represent metastasis from intracranial tumors or less commonly, de novo formation^4,5^. To date, comprehensive genomic characterization of gliosarcomas remains vastly under-reported, compared to gliobastomas.

In this study, we report a patient with a spinal cord gliosarcoma with prior history of a low-grade brain glioma treated with adjuvant chemoradiation. Whole-exome sequencing (WES) of the resected spinal tumor revealed the first case of a hypermutator phenotype to be described in gliosarcoma.

## Results

### Case description

**Fig. 1:**
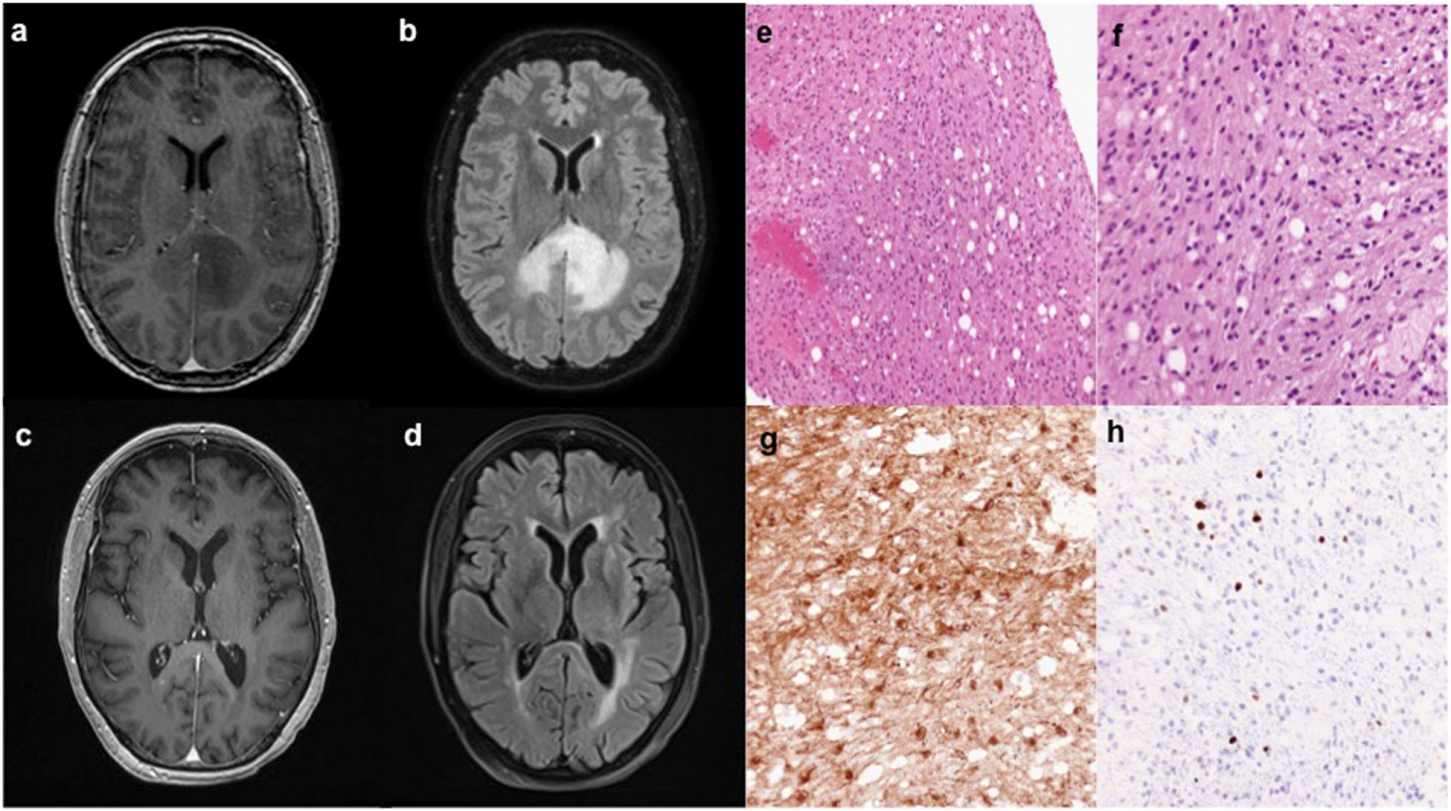
Imaging and histopathology of the low-grade glioma. **a** Representative axial slices of T1-weighted MRI after contrast administration and (**b**) T2-weighted FLAIR obtained prior to stereotactic brain biopsy show a predominantly non-enhancing lesion within the deep right parietal lobe with small areas of patchy enhancement that is crossing the corpus callosum with associated edema. Repeat imaging obtained nearly 2 years after biopsy and completion of adjuvant chemoradation demonstrates (**c**) stable foci of small nodular enhancement on T1-weighted MRI after contrast administration with (**d**) minimal associated edema on T2-weighted FLAIR. **e**, **f** An infiltrating astrocytoma without necrosis or vascular proliferation is seen on hematoxylin & eosin staining. **g** Immunohistochemistry for GFAP-positive tumor cells is shown and (**h**) the Ki-67 proliferative index was ~3%.

A 65-year-old female presented to an outside institution with several weeks of headaches and acute worsening of progressive numbness in her right lower extremity. Her past medical history was only significant for basal cell carcinoma of the left nasal ala, resected 5 years prior. Her family history was notable for breast cancer of middle-aged onset in her sister, mother, and grandmother but no prior diagnosis of a familial cancer predisposition syndrome. Her symptoms prompted magnetic resonance imaging (MRI) of the brain, which revealed a predominantly non-enhancing right parietal lesion with patchy enhancing foci extending to the splenium of the corpus callosum (Fig. 1a, b). Further imaging with computed tomography (CT) of the chest, abdomen, and pelvis did not reveal a primary systemic tumor to suggest a diagnosis of brain metastasis. She subsequently underwent stereotactic biopsy of her lesion with pathology demonstrating a glial neoplasm, consistent with World Health Organization (WHO) grade II astrocytoma (Fig. 1e–h). The Ki-67 index was 1–3% and immunohistochemical staining for *TP53* was positive. Further molecular testing was negative for the *EGFR* variant III, *IDH1* mutation, and *MGMT* promoter methylation. She was subsequently referred to our institution for further adjuvant treatment, where she underwent adjuvant radiation therapy and temozolomide treatment, as well as use of tumor-treating fields (TTF) with the Optune device (Novocure, Jersey, U.K.). A brain MRI obtained several months after completion of chemoradiation and TTF showed radiographic response with stable small foci of enhancement and drastic reduction in perilesional edema (Fig. 1c, d). Clinically, the patient was stable with moderate cognitive deficits and requirement of mild assistance with activities of daily living.

**Fig. 2:**
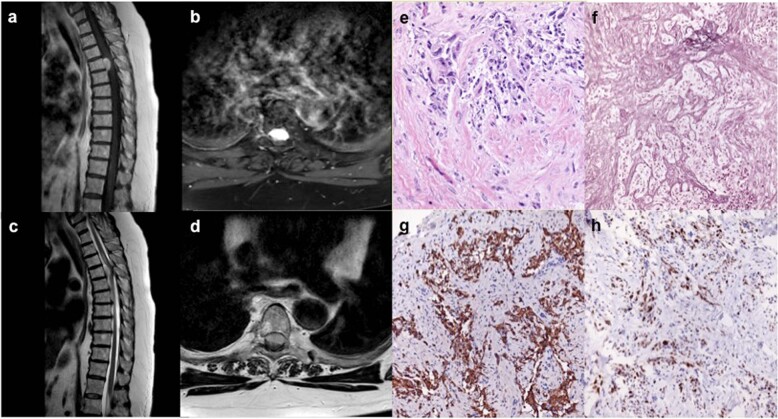
Imaging and histopathology of the gliosarcoma. **a** Representative sagittal and (**b**) axial slices from T1-weighted MRI after contrast administration show a homogeneously enhancing intradural, extramedullary lesion anterior to the spinal cord at the T4 level with associated cord compression seen on (**c**) sagittal and (**d**) axial slices on T2-weighted MRI. **e** Biphasic architecture is seen on hematoxylin & eosin staining, including (**f**) reticulin rich sarcomatous regions, interspersed with (**g**) GFAP-positive glial regions, and (**h**) an elevated Ki-67 proliferative index of ~10–15%.

Six months after completion of chemotherapy, she presented with increasing gait instability of two weeks duration and worsening thoracic back pain, localized between her shoulder blades. MRI of her thoracic and lumbar spine was obtained, demonstrating a homogenously enhancing dural-based, extramedullary lesion anterior to the spinal cord at T4 with moderate cord compression, causing edema extending from T2 to T7 (Fig. 2a–d). She was neurologically intact on examination other than brisk lower extremity reflexes, allodynia at the T4 level, and baseline right-sided hemibody numbness. Imaging findings were consistent with spinal meningioma, and the patient was admitted with plans for elective, inpatient surgery. However, two days after admission, she developed acute weakness in her bilateral lower extremities and subsequently was taken to the operating room for urgent resection of her tumor, which was removed in its entirety. Intra-operatively, the tumor was noted to be dural-based with a well-defined plane between tumor and the dura as well as the pia of the spinal cord. Motor evoked and somatosensory evoked potentials were improved at the end of the case, compared to baseline levels. Final pathology from the resected specimen was WHO IV gliosarcoma with biphasic tissue architecture, comprised of prominent GFAP-positive glial regions admixed with GFAP-negative spindled areas containing reticulin fibers (Fig. 2e–h). The Ki-67 index was elevated >10–15% and further routine molecular testing demonstrated *MGMT* promoter methylation but was otherwise negative for *IDH1* mutation and *ATRX* loss.

The patient was subsequently discharged to rehabilitation and exhibited some improvement in lower extremity strength, albeit remained non-ambulatory and required significant assistance with activities of daily living. Further treatment with adjuvant chemoradiation was considered but given the unfavorable prognosis from new gliosarcoma diagnosis and poor performance status, she elected to undergo hospice care without further imaging or treatment and subsequently expired one month after surgery.

### Genomic analysis

**Fig. 3:**
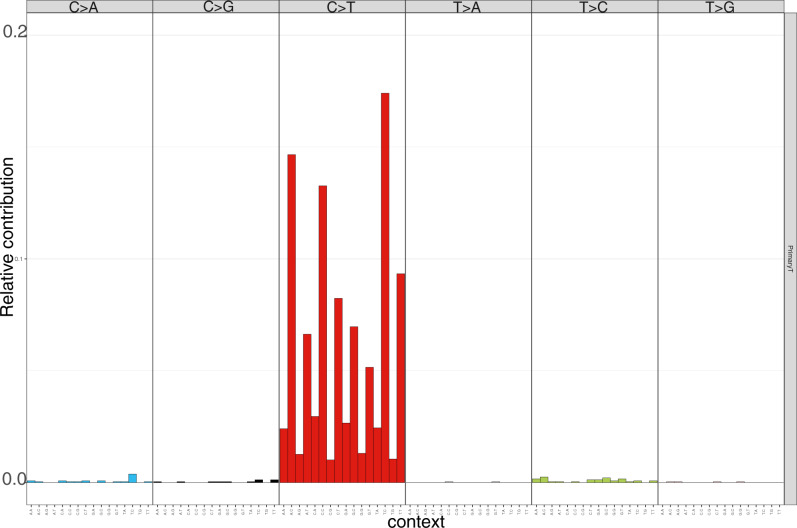
Mutational signature plot of SNV in the resected spinal tumor specimen. There was an overwhelming prevalence of C>T, with a SNV transition ratio of 96%, consistent with a hypermutated phenotype.

WES was performed on the gliosarcoma specimen, together with matching normal blood, to identify somatic single nucleotide variations (SNVs), insertions/deletions (INDEL), and copy number variations (CNV). The protocol for sequencing and analysis was in accordance with our previously described methods^6^. We detected 2372 somatic SNVs with a 96% C>T transition ratio (Fig. 3), consistent with a hypermutated phenotype. There were disease-associated alterations in key oncogenes, *PTEN*, *PIK3R1*, and *ATM*. Furthermore, there were key mutations in genes associated with the DNA mismatch repair (MMR) pathway, predicted to be deleterious based on scores from the following computational prediction tools: SIFT^7^, Polyphen2 HDIV Prediction^8^, Polyphen2 HVAR Prediction^8^, FATHMM^9^, MetaSVM^10^, and MutationTaster^11^. These included deleterious missense (p.P656L) and stop-gain (p.W105X) mutations in *MSH6* and a stop-gain mutation in *MSH3* (p.R727X). Additionally, there was a heterozygous germline variant in *MSH2* (p.H46Q) of unknown significance. Somatic CNV analysis revealed amplifications of chromosomes 7, 20, and X, deletions on chromosomes 10, 13, 17, and focal CNV events on chromosomes 1 and 5. 27% of the genome was altered by CNV and/or loss-of-heterozygosity events.

Given the relationship between microsatellite instability (MSI) and MMR deficiency in systemic cancers^12^, we performed further computational analysis with MSIsensor, an algorithm that predicts the microsatellite status of tumors from WES data^13^. The calculated MSI-score was 0.15, indicative of microsatellite stability per the tool guidelines, which uses a cut-off score of 3.5 for MSI.

## Discussion

Gliosarcomas have a high predilection for metastasis to both visceral sites as well as the CNS, via hematogenous and leptomeningeal spread^14^. As such, most cases of gliosarcomas involving the spinal cord have been secondary to metastasis from intracranial origin^4,15–19^. In contrast, primary gliosarcomas of the spinal cord are less common^5,20–22^ and are vastly outnumbered by glioblastoma when analyzing primary malignant gliomas of the spinal cord^3^. Interestingly, these tumors may present as intramedullary^15,23^, as well as intradural, extramedullary lesions^14,22^, regardless of primary or metastatic etiology. Similar to their intracranial counterparts, spinal cord gliosarcomas demonstrate avid and typically homogenous enhancement, which in cases of intradural, extramedullary location, may be mistaken for a meningioma, as demonstrated in our patient. A prior history of a primary brain tumor must raise suspicion for an alternative diagnosis and highlights the need for surgical intervention to aid in definitive tissue diagnosis.

It remains unclear whether the gliosarcoma in our patient represented metastasis from her intracranial astrocytoma or de novo formation of a new primary tumor. This may have been addressed with additional WES data or targeted molecular testing from her original brain tumor, but unfortunately, inadequate tissue was available for further genomic studies. Additionally, further molecular testing of her brain tumor would have been helpful, as *IDH*-wildtype astrocytomas with grade II histology may exhibit survival outcomes akin to IDH-wildtype glioblastoma^24,25^. Based on recommendations from the Consortium to Inform Molecular and Practical Approaches to CNS Tumor Taxonomy (cIMPACT-NOW)^26^, molecular testing to assess for *EGFR* amplification, chromosome 7 gain/chromosome 10 loss, and *TERT* promoter mutations to diagnose a diffuse astrocytic glioma, *IDH*-wildtype, with molecular features of glioblastoma, WHO grade IV could have been performed to predict increased biological aggression and perhaps tailor clinical management^27,28^. The existing literature of metastatic gliosarcomas to the spinal cord have all been from primary intracranial gliosarcoma, so the notion that our patient’s gliosarcoma represented metastasis, despite the difference in WHO grade from her initial tumor, would be unique. There have been few reports of progression of low-grade gliomas into intracranial gliosarcomas, in instances with and without adjuvant chemoradiation^29–31^. These studies confirmed metastatic origin through single nucleotide polymorphism (SNP) arrays and fluorescence in-situ hybridization (FISH) for chromosomal analysis, but to date, comprehensive sequencing to delineate the genetic changes that may underlie either malignant progression into gliosarcomatous pathology or de novo spinal cord gliosarcoma formation have not been described.

In recent years, the prevalence of hypermutagenesis has been described across a variety of cancers, including gliomas^32–34^ and particularly after therapy-driven changes in tumor evolution at the time of recurrence. In particular, treatment with temozolomide may induce mutations in the MMR pathway, leading to therapeutic resistance and acquisition of the hypermutated phenotype^35,36^. Strikingly, we found a hypermutated phenotype in our patient’s specimen, including mutations in MMR genes, *MSH3* and *MSH6*, which previously have not been associated with gliosarcoma pathology. Likewise, the abundance of C>T transitions detected within the somatic SNVs of our patient’s tumor was consistent with a MMR deficient hypermutated phenotype. We further explored the possibility of MSI, particularly given the strong correlation between MSI and MMR deficiency in systemic cancers that may serve as biomarkers for response to immunotherapies^37–40^. Utilizing the MSIsensor computational tool, we did not detect MSI despite the hypermutator phenotype of our patient’s tumor, a result consistent with prior studies failing to reveal an association between MSI and temozolomide-mediated hypermutagenesis in glioblastoma^36,41^. Taken together, these findings are consistent with a hypermutated phenotype secondary to temozolomide therapy in our patient’s gliosarcoma. Unfortunately, the WES data were not available in a timely enough manner to guide further treatment for our patient, such as the possibility of immunotherapy. Considering the clinical timeline of temozolomide treatment for her initial intracranial glioma but prior to spinal gliosarcoma diagnosis, this result also supports the notion of gliosarcomatous transformation from her lower grade glioma, rather than de novo tumor formation. We acknowledge that the inability to perform further genetic testing of her brain tumor is a key limitation of this study. Lastly, there are few reports of comprehensive genome sequencing of gliosarcomas, which have described key somatic mutations in known oncogenes including *TP53, PTEN, RB1*, and *NF1* as well as amplification events of *EGFR, CDK4/6, PDGFRA, MDM2, AKT1*, and *MET*^42–44^. However, this case represents the first report of hypermutagenesis within gliosarcoma and raises the possibility that acquisition of this genotype may be a mechanism of gliosarcomatous transformation from lower grade tumors.

We considered whether our patient’s gliosarcoma arose de novo with a predisposition from her detected germline *MSH2* variant. Sa et al identified a subset of treatment-naïve gliomas with de novo hypermutagenesis that had not been previously exposed to chemoradiation at the time of diagnosis^45^. Notably, these patients demonstrated germline mutations of MMR associated genes, including *MSH2, MSH3, MSH6*, and *MLH3* and relevant family histories of various cancers, together suggestive of diagnosis of Lynch syndrome. Likewise, gliosarcoma has been described in Turcot syndrome^46^, a condition characterized by familial predisposition to colorectal and brain cancers, secondary to germline mutations of the MMR genes^47^. In contrast, our patient’s germline variant in *MSH2* has been previously characterized as a benign variant, reported in individuals with colorectal, prostate, and ovarian cancers but not meeting clinical criteria for Lynch or Turcot syndrome or showing strong evidence for causality^48,49^. Furthermore, the lack of a second hit for *MSH2* supports the notion of our patient’s variant as benign. As such, it remains unlikely that our patient’s germline *MSH2* variant contributed to de novo tumor formation and/or acquisition of a hypermutator phenotype.

In summary, we describe the first case of a hypermutator phenotype associated with a gliosarcoma, characterized by somatic mutations in known MMR genes and consistent with temozolomide-mediated hypermutagenesis. Based on patterns of the described literature, it is more likely that our patient’s spinal cord gliosarcoma represents malignant progression and metastasis from her previous low-grade brain astrocytoma. As gliosarcoma remains a rare entity within primary malignant CNS tumors, even more so within primary spinal cord tumors, further comprehensive genomic characterization is needed to understand the genetic factors that can lead to malignant transformation from lower grade gliomas, as well as de novo formation of gliosarcomas.

## Methods

### Patient approval

This study was approved by Yale University’s Human Investigations Committee and Human Research Protection Program. Written informed consent was obtained from the parents of the patient. Specimens from resected tumors were evaluated microscopically by a board-certified neuropathologist.

### WES and analysis

Genomic DNA from the resected tumor specimen and blood were isolated, and exome capture was performed with IDT xGen Exome Research Panel v1 with the additional spike-in regions of ~620 kb of RefGene coding regions. Sequencing of the captured regions was performed at Yale Center for Genome Analysis (YCGA) using Illumina NovaSeq6000 with 2 × 100 bp reads. Mean coverage of 152.4x and 230.6x was achieved for normal and tumor, respectively. Pre-processing of the raw data, including alignment, PCR duplicate identification, re-alignment around INDELs, and base quality score recalibration was performed “GATK Best Practices” guidelines (GATK v4.1.8.1). Somatic variant calling for SNV/INDELs together with CNV analysis and annotation was performed as previously described in reports from our institution^50^. Microsatellite status was determined with MSIsensor, a publically available computational algorithm designed to predict microsatellite stability based on WES data^13^.

### Reporting summary

Further information on research design is available in the Nature Research Reporting Summary linked to this article.

## Supplementary information

REPORTING SUMMARY

## Data Availability

The data generated and/or analyzed during the related study are described in the figshare metadata record: 10.6084/m9.figshare.13347557^51^. The whole-exome sequencing data of the resected spinal tumor are shared openly in the European Genotype-phenotype Archive under accession https://identifiers.org/ega.study:EGAS00001004864^52^.
