# Supplementary material for: Hypermutated phenotype in gliosarcoma of the spinal cord
Source: NPJ Precis Oncol. 2021 Feb 12;5:8. doi: 10.1038/s41698-021-00143-w (PMC7881101; doi:10.1038/s41698-021-00143-w)
Supplement: Supplementary file 1 — REPORTING SUMMARY [file 41698_2021_143_MOESM1_ESM.pdf]

nature research

Corresponding author(s): Christopher Hong

Last updated by author(s): Oct 13, 2020

Reporting Summary

Nature Research wishes to improve the reproducibility of the work that we publish. This form provides structure for consistency and transparency in reporting. For further information on Nature Research policies, see our Editorial Policies and the Editorial Policy Checklist.

Statistics

For all statistical analyses, confirm that the following items are present in the figure legend, table legend, main text, or Methods section.

n/a	Confirmed	
☒	☐	The exact sample size (n) for each experimental group/condition, given as a discrete number and unit of measurement
☒	☐	A statement on whether measurements were taken from distinct samples or whether the same sample was measured repeatedly
☒	☐	The statistical test(s) used AND whether they are one- or two-sided<br>*Only common tests should be described solely by name; describe more complex techniques in the Methods section.*
☒	☐	A description of all covariates tested
☒	☐	A description of any assumptions or corrections, such as tests of normality and adjustment for multiple comparisons
☒	☐	A full description of the statistical parameters including central tendency (e.g. means) or other basic estimates (e.g. regression coefficient) AND variation (e.g. standard deviation) or associated estimates of uncertainty (e.g. confidence intervals)
☒	☐	For null hypothesis testing, the test statistic (e.g. F, t, r) with confidence intervals, effect sizes, degrees of freedom and P value noted<br>*Give P values as exact values whenever suitable.*
☒	☐	For Bayesian analysis, information on the choice of priors and Markov chain Monte Carlo settings
☒	☐	For hierarchical and complex designs, identification of the appropriate level for tests and full reporting of outcomes
☒	☐	Estimates of effect sizes (e.g. Cohen's d, Pearson's r), indicating how they were calculated

Our web collection on statistics for biologists contains articles on many of the points above.

Software and code

Policy information about availability of computer code

Data collection	Genomic DNA from the resected tumor specimen and blood were isolated, and exome capture was performed with IDT xGen Exome Research Panel v1 with the additional spike-in regions of ~620 kb of RefGene coding regions. Sequencing of the captured regions was performed at Yale Center for Genome Analysis (YCGA) using Illumina NovaSeq6000 with 2x100 bp reads.
Data analysis	Gene variants detected from WES were assigned scores based on the following computational prediction tools: SIFT, Polyphen2 HDIV Prediction, Polyphen2 HVAR Prediction, FATHMM, MetaSVM, and MutationTaster. Microsatellite status was determined with MSIsensor, a publically available computational algorithm designed to predict microsatellite stability based on WES data.

For manuscripts utilizing custom algorithms or software that are central to the research but not yet described in published literature, software must be made available to editors and reviewers. We strongly encourage code deposition in a community repository (e.g. GitHub). See the Nature Research guidelines for submitting code & software for further information.

Data

Policy information about availability of data

All manuscripts must include a data availability statement. This statement should provide the following information, where applicable:

- Accession codes, unique identifiers, or web links for publicly available datasets
- A list of figures that have associated raw data
- A description of any restrictions on data availability

Data generated during the current study are available from the corresponding authors upon request. Accession codes will be provided upon acceptance of the manuscript.

Field-specific reporting

Please select the one below that is the best fit for your research. If you are not sure, read the appropriate sections before making your selection.

☒ Life sciences ☐ Behavioural & social sciences ☐ Ecological, evolutionary & environmental sciences

For a reference copy of the document with all sections, see nature.com/documents/nr-reporting-summary-flat.pdf

Life sciences study design

All studies must disclose on these points even when the disclosure is negative.

Sample size	Case report, n=1
Data exclusions	No data excluded
Replication	Not applicable
Randomization	Not applicable
Blinding	Not applicable

Reporting for specific materials, systems and methods

We require information from authors about some types of materials, experimental systems and methods used in many studies. Here, indicate whether each material, system or method listed is relevant to your study. If you are not sure if a list item applies to your research, read the appropriate section before selecting a response.

Materials & experimental systems

n/a	Involved in the study	
☒	☐	Antibodies
☒	☐	Eukaryotic cell lines
☒	☐	Palaeontology and archaeology
☒	☐	Animals and other organisms
☐	☒	Human research participants
☒	☐	Clinical data
☒	☐	Dual use research of concern

Methods

n/a	Involved in the study	
☒	☐	ChIP-seq
☒	☐	Flow cytometry
☒	☐	MRI-based neuroimaging

Human research participants

Policy information about studies involving human research participants

Population characteristics	Case report, n=1
Recruitment	Patient consented to genomic analysis of tumor and normal tissues
Ethics oversight	Institutional Review Board of Yale University

Note that full information on the approval of the study protocol must also be provided in the manuscript.
